# The development of the Promoting Independence in Dementia (PRIDE) intervention to enhance independence in dementia

**DOI:** 10.2147/CIA.S214367

**Published:** 2019-09-10

**Authors:** Lauren Yates, Emese Csipke, Esme Moniz-Cook, Phuong Leung, Holly Walton, Georgina Charlesworth, Aimee Spector, Eef Hogervorst, Gail Mountain, Martin Orrell

**Affiliations:** 1 Institute of Mental Health, University of Nottingham, Nottingham, UK; 2 Division of Psychiatry, University College London, London, UK; 3 Department of Psychological Health and Well-Being, Faculty of Health Sciences, School of Health and Social Work, University of Hull, Hull, UK; 4 Department of Applied Health Research, University College London, London, UK; 5 Department of Clinical, Educational and Health Psychology, University College London, London, UK; 6 National Centre for Sports and Exercise Medicine, Loughborough University, Loughborough, UK; 7 Centre for Applied Dementia Studies, University of Bradford, Bradford, UK

**Keywords:** self-management, public patient involvement, behavior change, manual, cognitive impairment

## Abstract

**Objective:**

Support after a diagnosis of dementia may facilitate better adjustment and ongoing management of symptoms. The aim of the Promoting Independence in Dementia (PRIDE) study was to develop a postdiagnostic social intervention to help people live as well and as independently as possible. The intervention facilitates engagement in evidence-based stimulating cognitive, physical and social activities.

**Methods:**

Theories to promote adjustment to a dementia diagnosis, including theories of social learning and self-efficacy, were reviewed alongside self-management and the selective optimization model, to form the basis of the intervention. Analyses of two longitudinal databases of older adults, and qualitative analyses of interviews of older people, people with dementia, and their carers about their experiences of dementia, informed the content and focus of the intervention. Consensus expert review involving stakeholders was conducted to synthesize key components. Participants were sourced from the British NHS, voluntary services, and patient and public involvement groups. A tailored manual-based intervention was developed with the aim for this to be delivered by an intervention provider.

**Results:**

Evidence-based stimulating cognitive, physical, and social activities that have been shown to benefit people were key components of the proposed PRIDE intervention. Thirty-two participants including people with dementia (n=4), carers (n=11), dementia advisers (n=14), and older people (n=3) provided feedback on the drafts of the intervention and manual. Seven topics for activities were included (eg, “making decisions” and “getting your message across”). The manual outlines delivery of the intervention over three sessions where personalized profiles and plans for up to three activities are developed, implemented, and reviewed.

**Conclusion:**

A manualized intervention was constructed based on robust methodology and found to be acceptable to participants. Consultations with stakeholders played a key role in shaping the manualized PRIDE intervention and its delivery. Unlike most social interventions for dementia, the target audience for our intervention is the people with dementia themselves.

## Background

The UK government has placed emphasis on the development of accessible, high-quality specialist services to support the growing number of people with dementia and their supporters, for example Challenge on Dementia 2020.^1^,^2^ Receipt of support soon after diagnosis can facilitate better adjustment and ongoing management of dementia.^3^ In the UK, “dementia adviser” services can be a key aspect of postdiagnostic care to offer information, advice, and help to facilitate access to local services. Support may help people remain at home in their community for longer, may delay or reduce residential care placement, and can help people and their carers to establish a positive narrative around their life post-diagnosis.^4^

People with dementia may reduce their daily activities and become less independent, not only due to neurological decline, but also because of “excess disability” rooted in stigma and demoralization, a sense of loss of autonomy and confidence, and restricted perceptions of what they can do.^5^ People with dementia report challenges to creating a positive narrative around “life with dementia” such as other people behaving in a condescending or overprotective way.^6^ Feeling “devalued” in the wake of diagnosis is commonly cited as a source of concern for people with dementia, particularly with others being aware of their diagnosis.^7^ Narratives of deficit fail to reflect the agency people with dementia can enact to shape their social worlds. This can be mitigated by social capital, personal and cultural beliefs, and the responses of others.^8^ Studies focused on enhancing the lives of people with dementia suggest that a supportive and inclusive environment is crucial in moving forward postdiagnosis, sustaining identity, and continuing to live a life with meaning and value.^9^,^10^

The Promoting Independence in Dementia (PRIDE) program aims to better understand the factors associated with cognitive decline and “excess disability” and to design and evaluate an evidence-based approach to maintaining independence in people with mild dementia (https://www.institutemh.org.uk/research/projects-and-studies/current-studies/protect/246-the-pride-study). Expanding on the brief overview of intervention development in the feasibility assessment protocol,^11^ this article describes the underlying theory and proposed mechanisms of change for the PRIDE intervention, a 3-session, manualized, postdiagnostic social intervention to help people with dementia live as well and as independently as possible in the community through engagement in cognitive, physical, and social activities.

### Aims

The aim of the intervention development phase of PRIDE was to draft and refine a manual for people with mild dementia to support engagement in cognitive, social, and physical activities. The intervention strategies in the manual include behavior change strategies (goal-setting, problem-solving, and decision-making) for behavior change, case illustrations for social learning, and information provision for knowledge acquisition.

## Methods

The Medical Research Council (MRC) is a UK-based independent advisory board set up to support scientific research into human health, and is the author of a number of guidance texts designed as references for the scientific community. The guidance for complex interventions^12^ outlines four key stages of the development and evaluation process: 1. Development, 2. Feasibility/Piloting, 3. Evaluation, and 4. Implementation. The intervention development for this study was based on stages one and two. The development phase involves identifying existing evidence, developing theories and modeling process and outcomes, and the feasibility/piloting. This article describes the development and piloting stages.

### Examination of existing literature (1)

Existing theories, models, and frameworks for well-being in later life and dementia including self-management, selective optimization and compensation, social network and learning theories, and self-efficacy theory were explored. This informed the preliminary contents and focus of the intervention, along with key policy documents on psychological and social interventions in early-stage dementia.

### First stakeholder consultation/drawing together epidemiological and qualitative work (2)

Twenty-nine expert stakeholders were invited to take part in formal meetings on six occasions to help develop the intervention. Additionally, less formal smaller meetings and teleconferences were held in between to further develop what would eventually be included. This work focused on evaluating and choosing which existing theories and literature were appropriate to the social intervention. The workgroup included Patient and Public Involvement (PPI) representatives (n=5), consultants of old age psychiatry (n=3), clinical psychology (n=4), occupational therapy (n=1), health psychology (n=2), health economists (n=2), epidemiologists (n=4), general practitioners (n=1), postdoctoral researchers (n=4), and PRIDE PhD students (n=3).

The intervention draws on other complementary strands of the overall project in which it is embedded. The English Longitudinal Study of Ageing (ELSA) is a database of a representative cohort of women and men 50–100 years of age in England (n>11,000) and well suited to the investigation of processes related to changes in cognition in older people.^13^ Memory, executive function, physical and mental health, lifestyle, social and civic participation, and psychosocial factors amongst others are assessed every 2 years. This database was used to track changes over time as well as associations between factors as predictors of cognitive decline and the impact of such decline on future health, family connections, and social participation. Second, qualitative work focused on social discourses of dementia with a particular focus on independence and the lived experiences of people with memory problems across the dementia trajectory. To do this, two in-depth open-ended, semistructured interviews were conducted 18 months apart with a cohort of 120 individuals ranging from those having no memory problems to those 2-years postdiagnosis.^14^ Transcript data were thematically analyzed.

### First draft of the manual (3)

A draft of the manual was developed based on stages one and two of the framework.

### Second stakeholder consultation (4)

Draft one of the manual was presented to a number of stakeholder consultation groups made up of individuals with dementia, older adults, intervention providers, and carers who had not formed part of the main working group and a second draft of the manual was created.

### Second draft of manual (5)

A final manual was created based on the work carried out.

**Figure 1:**
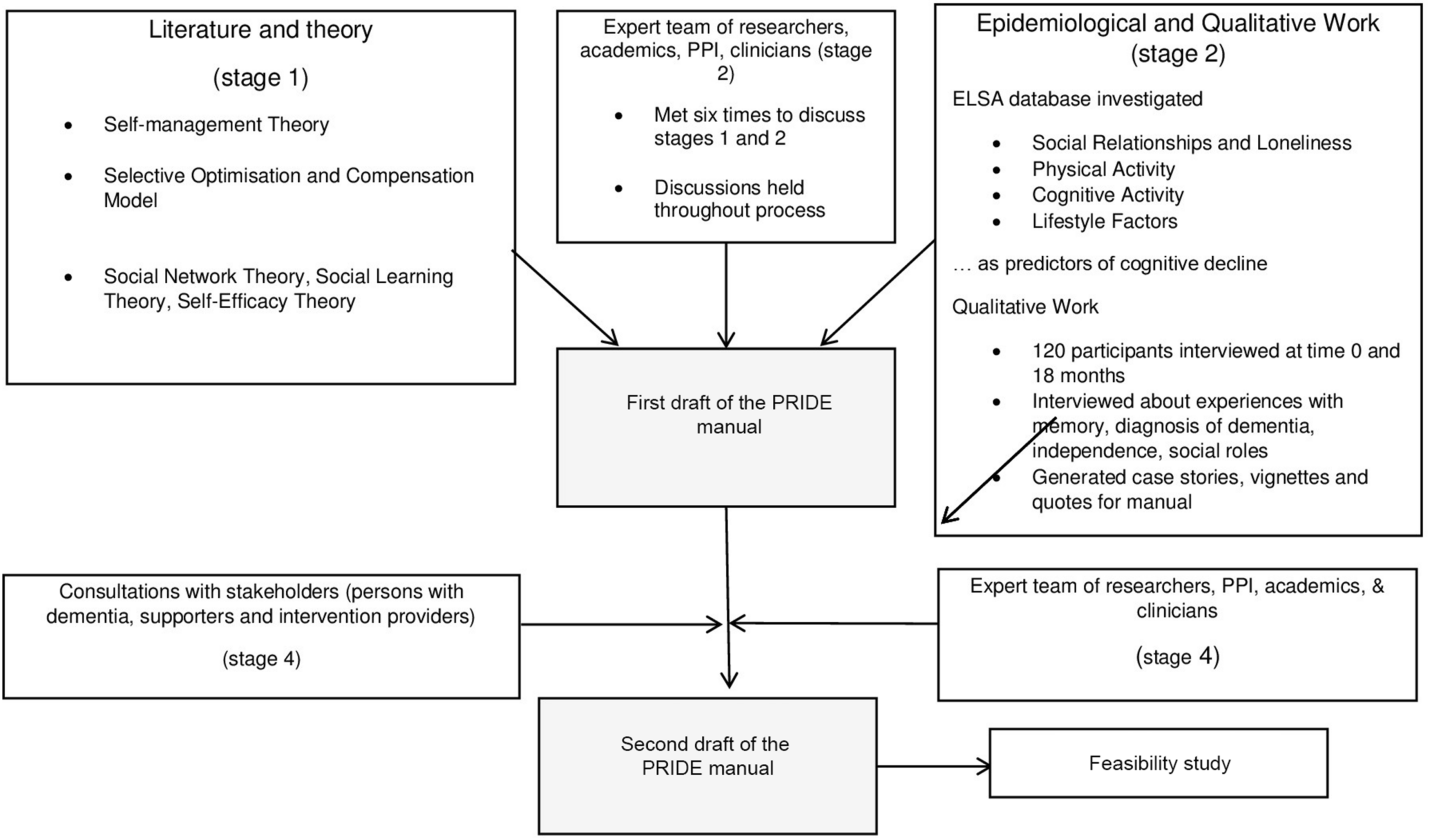
Overview of development phases of PRIDE intervention and manual drafting within the MRC framework. **Abbreviations:** PPI, Public Patient Involvement; ELSA, English Longitudinal Study of Ageing; PRIDE, Promoting Independence in Dementia; MRC, Medical Research Council.

Please see Figure 1 for an overview of development phases of the PRIDE intervention and manual drafting.

### Interview sample and recruitment

An opportunistic sample of project stakeholders known to the team (eg, university PPI groups, collaborating dementia cafes) were recruited to consult on the first draft of the manual. Individual participants gave permission to be contacted by researchers either by telephone or by email to discuss the study and arrange visits.

### Ethical considerations

This was a consultative patient and public activity and did not collect participant data; therefore, ethical consent was not required.^15^ Potential participants that were approached were part of a pool of people already in contact with the research team including volunteer sector affiliates, existing dementia cafe attendees. All participants verbally agreed to participate in discussions with researchers. People with dementia were in the mild stages with a capacity to indicate their preference to take part or not.^16^ All participants were provided with a brief information sheet describing the nature of the consultation session. Printed or electronic copies of the first draft of the manual were sent out to participants prior to the consultation where possible to give participants time to familiarize themselves with the material.

Consultations were conducted on a one-to-one basis, in the form of group discussions, or via email. Researchers had a list of questions to refer to, and a formal topic guide was used covering topics including case stories, feasibility, navigation, and language used. The researcher noted comments during the discussion. Individual consultations typically lasted between 30 mins and 1 hr, and group consultations around an hour.

### Intervention delivery

Running concurrently with the intervention development (although not a topic for this article), the delivery of the intervention was considered and developed. It was decided in the interests of generalizability and variation in local services, intervention providers could be health or voluntary sector professionals (eg, nurses and dementia advisors) working with people with dementia. The PRIDE team developed a training guide for intervention providers with supplementary information and reflective questions to consolidate information learned from the training session. The treatment integrity model was used to ensure the delivery of the intervention as intended, for example providing detailed descriptions of intervention components in accordance with recent guidelines and applying standardized procedures.^17^

## Results

### Examination of existing literature (1)

#### Self-management theory

Self-management interventions are widely used in the treatment of chronic conditions, such as asthma and diabetes.^18^ Self-management engages the individual in learning to manage their condition and to identify solutions according to their specific needs.^19^ Reported benefits of the approach include increased knowledge, increased sense of control over life with the condition,^20^ enhanced self-efficacy,^21^ and improvement of quality of life, clinical outcomes, and health service use.^22^ Developing strategies such as problem-solving, decision-making, selecting and making use of resources, making informed choices about care in partnership with health care professionals, and making steps to implement changes are key elements of self-management.^23^,^24^ Having these self-management strategies available and being in a position to implement them may help persons with dementia tackle feelings of being undermined or devalued as described in Sterin^6^ and later Langdon’s work.^7^ There have been few applications of this approach in interventions in dementia. However, self-management could offer the opportunity for inclusion, as the person with dementia adopts an active role in everyday coping with their condition.^25^,^26^

The opportunity for autonomy and participation in decision-making postdiagnosis is important to create a positive narrative about dementia for people with dementia and their supporters. However, as the person’s decision-making capacity may fluctuate and deteriorate over time,^27^ supporters often become more involved in decision-making even in the early stages of dementia.^28^ Carers may increasingly lead on decisions about risk assessments (eg, personal safety), practical tasks (eg, finances), and upkeep of health and social care (eg, medical treatments).^28^ This shift can threaten the person with dementia’s sense of autonomy.^29^ Other research has reported that people with dementia and carers consider decision-making and shared decisions as important to autonomy but did not often consider this in everyday life.^30^ Furthermore, while people with dementia wanted to sustain their involvement in daily decision-making processes, they also had confidence in their carer (relatives or friends) to make the right decision for them if necessary.

Self-management techniques and everyday decision-making can be used to help people with dementia take control of their care and activities. Elements of self-management were incorporated into the PRIDE intervention in order to enable the person with dementia to have an active role in the management of the condition, in which they 1) define the level and type of support they would like from those around them, 2) pursue specific goals they have chosen to help them to live well, 3) continue to be part of their community, 4) participate in meaningful and enjoyable activities and 5) explore strategies which may help them adapt to challenges they face.

#### Selective optimization with compensation (SOC) model

The decline in cognitive health which is symptomatic of dementia may compromise quality of life, independence, social connectedness, sense of purpose, functional recovery (eg, illness), and ability to cope with functional decline.^31^ The PRIDE intervention seeks to counter this by involving strategies to preserve cognitive health for as long as possible after diagnosis. The SOC model^32^ specifies that the extent to which losses in ability can be minimized is dictated by the interaction between the person’s internal states and capacities, the demands of their environment, and contextual opportunities they engage in. The model is embedded in the content of PRIDE in that the intervention encourages the person to exercise elective selection (choosing things they would like to do). The person then carries out optimization behavior (applying methods and available resources to achieve the things they have “selected”). Finally, when the person faces challenges in cognitive or functional capacity, they choose compensatory (alternative) strategies to ensure they can continue to do the things they would like to do.

#### Social network theory, social learning theory, and self-efficacy theory

Social network theory^33^ emphasizes the important role of social networks and relationships in the management of chronic conditions. In line with the social network theory, the PRIDE intervention includes topics such as participation in social activities and identification and development of the person’s social network. Cultivating a rich social environment can enhance self-esteem and enable people to better cope with stress.^34^ Social networks also offer resources and information which can be of tremendous benefit to the person with dementia and their carer.^4^ Social learning theory^35^ was important in the development of the content of the manual and the role of the dementia adviser. Vignettes or “case stories” were derived from earlier PRIDE interview data from people with lived experience of memory problems and dementia and qualitative studies.^36^–^39^ The role of the dementia adviser is to encourage the person and their supporter to reflect on these examples with respect to their own circumstances and behavior. In association with social learning theory, self-efficacy^40^ may be an important mechanism present in the PRIDE intervention. Having support from the dementia adviser/facilitator and a friend or family member, available tools and resources and developing strategies for everyday challenges and activities as part of the intervention may increase the person’s sense of being able to confidently accomplish meaningful self-defined goals through activities/actions.

### First consultation/development with key stakeholders (2)

Over the course of meetings, the PRIDE intervention was conceptualized and priority areas for the intervention content were identified. Literature described above was considered and debated. Studies examining protective/risk factors, such as loneliness, physical activity, and computer use, have also shown beneficial effects of these activities in early-stage dementia. For instance, ELSA data showed that people who stayed physically active after diagnosis had less cognitive decline.^41^ Using ELSA, we found that dementia risk was positively related to loneliness, fewer close relationships, and not being married later in life.^42^,^43^ Furthermore, marital status (eg, having a constant carer present) can facilitate uptake of activities.^44^ Social isolation and loneliness are also important factors for mental health and physical well-being generally and are therefore important considerations for an intervention focused on maintaining activities. Computer use was also found to be a protective factor against developing dementia or improving cognition.^45^,^46^ These data on tertiary prevention are woven into work with theories such as self-management (eg, the goal to continue doing social activities, such as being part of a walking group).

Anonymous interview data featuring in the manual were used either as a basis to form scenarios for the case stories, to supplement information resources (please see Box 1 for examples). We used these case stories to ensure that the contents and style of the manual reflected the current concerns of people with dementia, rather than experts deciding on their behalf. Online resources such as Alzheimer’s Society factsheets (www.alzheimers.org.uk) and National Health Service (NHS) Choices (https://www.nhs.uk/Conditions/Pages/hub.aspx) were used as references for information provided in the manual. The Practitioner Assessment of Network Type (PANT)^47^ and Circles of Support model^48^ were adapted as tools to facilitate discussion and mapping of the person with dementia’s support network in the “People and connections” section.

**Table 1:** Content of PRIDE manual, source from which information was derived/adapted and theoretical models and frameworks

PRIDE topics/process	Description of topic	References/evidence	Theory/mechanisms
Finding a balance	Helping the person to think about time and resources available/needed in order to get the most out of activities. How to adapt activities that have become or may become challenging Rest and relaxation Routines and reminders	Qualitative interviews	Self-management: selecting and making use of resources Self-efficacy theory^35^, ^40^ Selective Optimization with Compensation (SOC) model^32^
People and connections	Information and activities about how people can provide support to the person with dementia Thinking about how the person’s support network can be enhanced, and who might be around to support the person to do specific things Finding a balance in the amount of support that is provided to the person	The Practitioner Assessment of Network Type (PANT)^47^ Circles of Support model 48	Social network theory^33^ Self-management: selecting and making use of *social* resources
Keeping going	Introduction to the plan, do, review process including examples of completed worksheets Finding motivation to do activities and “keep going” Getting around as a practical consideration in planning and doing activities out in the community		Self-management: problem-solving strategies
Keeping mentally active	Information about mentally stimulating activities Examples of carrying on, doing more, and trying new mentally stimulating activities	Epidemiological data^13^ Cognitive Stimulation Therapy (CST) research and evidence, qualitative interviews	Use it or lose it
Keeping physically active	Information about physical activities Examples of carrying on, doing more, and trying new physical activities	Epidemiological data^13^ Qualitative interviews	
Keeping socially active	Information about socially stimulating activities Examples of carrying on, doing more, and trying new socially stimulating activities	Epidemiological data,^13^ research literature, qualitative interviews	Social network theory^33^
Making decisions	Information and activities to help the person with everyday decision-making Case stories and quotes to help the person reflect on their own situation Examples of challenges people experience when making decisions and problem-solving tips Examples of how people have effectively overcome challenges with decision-making	Qualitative interviews	Self-efficacy theory^35^,^40^ Self-management: decision-making
Getting your message across	Information and activities to help the person communicate with those around them Supportive relationships Case stories and quotes to help the person reflect on their own situation Examples of challenges in communication	Qualitative interviews	Social network theory^33^
What does it mean to be told you have dementia?	Information about receiving a diagnosis of dementia, managing worries, and sharing a diagnosis with others Case stories and quotes to help people reflect on their own situation	Qualitative interviews	Developing a positive narrative of life with dementia Social network theory^33^
Keeping healthy	Links to websites and resources about keeping physically healthy including general health, heart health, diabetes, lifestyle (eg, nutrition, weight management, sleep, worries), dental health, and smoking and drinking alcohol.	Web-based resources and fact sheets from organizations such as the Alzheimer’s Society, and National Health Service (NHS) Choices, University of Waterloo Living Well resources	Self-management: making informed choices about care and health
Plan, do, review process			Self-management: Plan: problem-solving, decision-making, selecting and making use of resources, making informed decisions about health and care Do: making steps to implement behavior change Review: problem-solving

**Box 1:** Case stories based and examples drawn from qualitative interviews and results of epidemiological findings

**Samuel** **and** **Rose** Samuel has dementia. His wife, Rose, takes care of a lot of things around the house. He describes how he feels about making decisions: “Quite happy to go along. My wife is a very good judge of character and I won’t interfere with that at all. If she says we’re going to have chops for dinner, I won’t argue because she’s such a good cook and there’s no point in talking about it any more.” – Samuel Samuel is happy for others to make decisions for him Decisions may be discussed, but when asked, Samuel often says to Rose; “That’s up to you. You do what you think” Samuel may be finding it difficult to make certain decisions	**Gloria** Gloria has always been a very independent lady and has lived on her own for a long time. She is reluctant to accept any support as she feels this will compromise her independence. “My independence is really important to me, and I know if someone came in and started telling me how I should run things or do things, I think I would certainly retaliate and not conform to anything they would want to do.” *–* Gloria Gloria has always made her own decisions. Gloria does not like other people interfering. Gloria doesn’t like asking for help. Others around Gloria may have tried to help, but Gloria has declined this.
**Hal’s Story: I have trouble with my hearing and my sight** “Hearing can be a worry. In a noisy place I will miss a lot of information or conversation that’s going on.” – Hal If you’re concerned about your hearing or vision, book an appointment to have your hearing and sight tested. High street opticians often offer both services. If you already have hearing aids it might be worth checking you have the correct batteries, or that your hearing aid isn’t broken. If you already have glasses, perhaps your prescription may need to be updated as sight can change. In group situations ask people to speak more loudly, clearly, or repeat what they are saying if you didn’t catch it the first time.	**Ben and Sade** Privacy – Some people prefer to keep information about their lives and health conditions private “We haven’t told the neighbours – there’s no need to. We hardly meet the neighbours, really. They’re not the sort of neighbours like we’re used to.” – Ben and Sade Worry or fear – Some people are worried about how others will react if they know about their diagnosis. They may feel like this about everyone, or just certain people.

The Dementia Engagement and Empowerment Project (DEEP) guidelines^49^,^50^ were followed to ensure information in the draft manual was presented in an accessible way for people with dementia, including the type of language used, formatting, and layout. In addition, other intervention manuals produced by members of the workgroup in conjunction with stakeholders (eg, Making a Difference 3)^51^ were used to inform the presentation of information in the PRIDE manual. No formal methods of analysis were performed at this stage in the project. The workgroup agreed that the intervention should adhere to the principles of person-centered care,^52^ enabling communication and relationship building between persons with dementia, their supporter or carer, and intervention provider.

Please see Table 1 for a summary of the intervention content and sources.

Decisions made:

- Existing literature and expert knowledge form the starting point for development work
- Epidemiological findings and qualitative work to be incorporated into intervention
- Manual should adhere to principles of person-centered care
- DEEP guidelines used

### First draft of the PRIDE manual (3)

#### Content of the PRIDE manual

Based on our evaluation of the literature and our own findings, the manual aimed to be a source of information, including case stories, and practical activities to complete with the advisor during the PRIDE sessions. The manual has a menu-based structure, embedding choice about content and allowing the person to tailor the intervention to their interests and desired outcomes. The intervention includes three “core” topics (“Finding a balance”, “People and connections”, and “Keeping going”) and seven optional topics to choose from (“Keeping mentally active”, “Keeping physically active”, “Keeping socially active”, “Making decisions”, “Getting your message across”, “What does it mean to be told you have dementia?”, and “Keeping healthy”). Recognizing the importance of acknowledging the lived experiences of people with dementia and the challenges they face in remaining independent and agentic,^8^ we used participants' examples drawn from the qualitative work package:

(eg, For the time being while I can still do a lot of things myself without too many problems, then I don’t want to have to depend on other people. I can’t just keep saying to my family, ‘Take me here, do this, do that’. I like being independent.)

Keeping mentally, physically, and socially active were featured as topics based on data from the longitudinal analyses of modifiable risk factors of cognitive decline and dementia.^41^–^46^

The workgroup decided that the “Keeping healthy” (eg, nutrition, heart health) topic should be concise, serving to signpost to useful resources and organizations, rather than attempting to provide comprehensive information. This would also circumvent inaccuracies stemming from changes in the provision of services and the need to tailor it locally. It was felt that people should consult with relevant health care professionals if they had any concerns, but that PRIDE’s role could be to encourage people to explore and reflect on general healthy living practices.

#### Structure of the intervention

**Figure 2:**
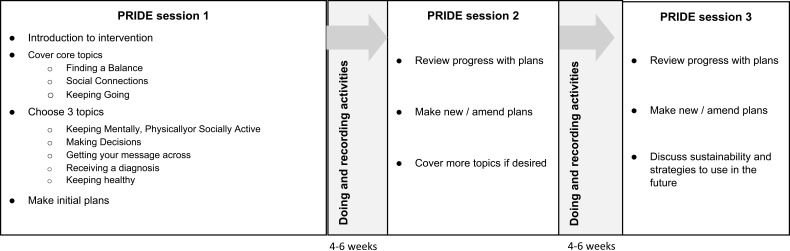
PRIDE intervention structure.

The intervention comprises three sessions with an intervention provider dementia adviser approximately 4 weeks apart (see Figure 2). The intervention provider helps the dyad plan activities, identify resources already available and signpost to resources that might be useful, review plans, and adjust them. Each session is expected to last between 1 and 1.5 hrs and is delivered in a place convenient for the person and their supporter. A supporter (eg, friend or family member) is involved alongside the person, but the intervention is primarily aimed at the person with dementia.

#### Plan, do, review process

The plan, do, and review steps are the basic steps involved in each session, as described below. This was planned to include behavior change techniques (BCTs) from existing literature^53^ such as goal-setting, action planning, self-monitoring of behavior/outcomes, and problem-solving. The planning aspect involves choosing an activity or action and considering the likely outcomes from a practical perspective with a strong intention driving people to carry them out (“do” element of PRIDE). The process is rounded up with a “review” guided by the dementia adviser that encourages the person to again apply problem-solving strategies to refine their plans, targeting any areas that may strengthen their intention to carry out the behavior if this was not possible in whole or in part between the sessions. Reviews may also culminate in the production of new plans.

#### PRIDE sessions

Session 1: In the first session, the intervention provider completes a profile of the person with dementia and discusses participants’ interests, current activities, and preferences. The intervention provider will discuss finding a balance with activities and social connections and introduce the “plan, do, review” process. The person will choose three of seven topics in the manual and put together plans to do an activity or action. In between sessions, the person will enact their plans, recording their efforts on “do” calendar-style worksheets provided.

Session 2: The person and their supporter will reflect with the intervention provider on whether they have enacted their plans, to what extent, and whether their plans require adjustment which is referred to as a “review”. The person may make more plans and discuss information and resources from topics they wish to cover. Between the second and final sessions, the person will enact their plans or actions and record them.

Session 3: In the third and final session, the person will “review” the implementation of their plans with the intervention provider. The session will also be focused on how the person and their supporter may take the information and skills they’ve learned from the program forward in the future in order to sustain independence and involvement in everyday activities and decisions.

Decisions made:

- Overall structural and content decided
- Qualitative data were utilized in the manual
- Findings from the longitudinal data (eg, keeping physically active) guided content and direction of manual
- “Keeping healthy” was to be signposted rather than detailed
- Plan, do, review process incorporated into the structure

### Consultations on first draft of PRIDE (4)

#### Sample

Thirty-two individuals agreed to participate in the consultations, 19 women (60%), nine men (28%), and four were unrecorded (12%). Of these, four (12.5%) had a diagnosis of dementia, 11 (35%) were carers; 14 (44%) were dementia advisors, and three were older adults or care staff (9%). Eight individuals (25%) were recruited via the Alzheimer’s Society, 12 (37%) from other voluntary organizations, six (19%) from memory cafes, three each (19%) from PPI groups and participants from the qualitative study. Twenty-three (72%) were sent the manual before the consultations. Notes taken by the researcher at the consultations were combined with feedback provided by participants via email and comments written in the manual. Comments were categorized by two researchers into feasibility and design issues, which were then used to generate action points for changes.

### Second draft of the PRIDE manual (5)

The action points (see Box 2) from the first consultations were carried out to create draft two of the manual, which will be tested in a feasibility study.

**Box 2:** Changes made in response to consultations

**Examples of changes made: structure**
In the first iteration, an overview of the programme and sessions in the form of a game board was included. Although participants said it was important to provide this information so that people would know what to expect throughout the intervention, they felt the design was “too busy” thus this was redesigned in the second draft. “The diagram is very nice and clear, but might be too much for people with dementia.” (Carer, memory café consultation) “Daunting, dementia adviser would be okay with it, better to break it down into sessions eg, page for start, page for session 2 etc.” (Dementia adviser, consultation group) A modular approach was considered with separate booklets for each topic, but ultimately rejected. “Easy and less daunting if it were split into booklets based on needs.” (Dementia adviser, consultation group) The initial version of the social connections mapping exercise had many different blank sections for the person to add detail. Some participants felt it could be disheartening for people “to realise how few people they have in their lives” if they were not able to fill in all of the “social map”; therefore the design was made simpler, with fewer boxes to fill in. “Support network: It is much too much. I was crying when I read this page. New friend? No, how to get new friends when you are old and living with dementia.” (Person with dementia, interview consultation) “If someone had hardly anyone in their support network, the section on this might be upsetting – to realise how few people they have in their lives.” (Carer, interview consultation)
**Examples of changes made: content**
A number of participants said the manual was too long and that this may be overwhelming for those using it in the sessions. “Not very user friendly as it has too much information. This will put them off straight away.” (Carer, memory café consultation) “Even if the manual is smaller (in length) it won’t get people to pick it up. They’ll put it down and won’t remember where it is.” (Dementia adviser, consultation group) Vignettes were initially labelled as “case stories”. However, this was not well received. “Case story sounds childish – case study is a term most people are familiar with.” (Person with dementia, email consultation) “Sounds like ‘case history’ – medical/professional sounding. ‘Personal story’ or ‘your story’. ‘Jill’s story’” (Dementia adviser, consultation group) The title of each vignette was changed as suggested so that it included the name of the character featuring in the scenario. For example, “Inge’s Story”. Some participants were concerned that the planning aspect of the intervention would not be suitable as it required cognitive skills, which tend to decline with dementia. “A lot of strategies for improvement are based around giving a person ‘homework’- to sit down and write things down, listing things and even searching online all the tasks requiring a lot of initiative, planning and organizational skills which are often affected most. (Dementia adviser, email consultation)” Furthermore, they pointed out that activities may not be suitable for people depending on their educational and work life background. “Planning and organizational skills and writing things down might be quite developed for the people of certain educational background but not for the people who worked in more manual jobs or have been retired for a long time and main hobbies were more practical – gardening, cooking, housework, sports etc.” (Dementia adviser, email consultation)
**Examples of changes made: miscellaneous**
Several participants felt that stipulating a supporter is required for the intervention would exclude those who might stand to benefit but who could not identify someone to participate alongside them. “It’s a shame you’re excluding people who don’t have a carer or friend who can attend with them. I’d be excluded as I don’t have a carer and all my friends are at work.” (Person with dementia, email consultation)

**Table 2:** Behavior change techniques embedded in PRIDE for assessment of fidelity

Aspect of PRIDE manual		Behavior change techniques coded using BCTTV1 53
Necessary information	Introduction	N/A
	Finding a balance	1.1 Goal-setting behavior 3.1 Social support unspecified 3.2 Practical social support 4.1 Instruction on how to perform the behavior 7.1 Prompts/cues 8.1 Behavioral practice/rehearsal 8.3 Habit formation
	People and connections	1.2 Problem-solving 3.1 Social support unspecified 4.1 Instruction on how to perform the behavior 5.3 Information about social and environmental consequences 6.1 Instruction on how to perform the behavior
	Keeping going	3.1 Social support unspecified 4.1 Instruction on how to perform the behavior
Tailored topics	Tailored topic 1 (Keeping mentally active)	1.2 Problem-solving 5.3 Information about social and environmental consequences 8.7 Graded tasks
	Tailored topic 2 (Keeping physically active)	1.1 Goal-setting behavior 1.2 Problem-solving 3.1 Social support unspecified 4.1 Instruction on how to perform the behavior 5.1 Information about health consequences 5.3 Information about social and environmental consequences 5.6 Information about emotional consequences 8.1 Behavioral practice/rehearsal 8.3 Habit formation 8.7 Graded tasks 9.1 Credible source 12.1 Restructuring the physical environment
	Tailored topic 3 (Keeping socially active)	1.2 Problem-solving 3.1 Social support unspecified 3.2 Social support practical 5.1 Information about health consequences 7.1 Prompts/cues
	Tailored topic 4 (Making decisions)	1.2 Problem-solving 3.1 Social support unspecified 3.2 Social support practical 5.3 Information about social and environmental consequences 6.1 Demonstration of behavior 9.1 Pros and cons
	Tailored topic 5 (Getting your message across)	1.2 Problem-solving 4.1 Instruction on how to perform a behavior 5.6 Information about emotional consequences 9.1 Credible source
	Tailored topic 6 (Receiving a diagnosis of dementia)	1.2 Problem-solving 3.1 Social support unspecified 4.1 Instruction on how to perform a behavior 5.3 Information about social and environmental consequences 5.6 Information about emotional consequences 6.1 Demonstration of behavior 9.1 Credible source
	Tailored topic 7 (Keeping healthy)	4.1 Instruction on how to perform a behavior 5.1 Information about health consequences 9.1 Credible source
Plan, do, and review	“Plan”	1.1 Goal-setting behavior 1.2 Problem solving 1.4 Action planning
	“Do”	2.3 Self-monitoring of behavior
	“Review”	1.2 Problem-solving 1.5 Review behavioral goal
Feedback and support	Feedback and support	2.2 Feedback on behavior 3.1 Social support unspecified 10.4 Social reward

**Notes:** Information provided in this table is from the PRIDE intervention framework which was used to develop PRIDE fidelity checklists (Walton, 2018).^54^

A number of issues were highlighted by those consulted, which were either addressed immediately and incorporated into the second draft of the manual. Minor amendments to the manual included simplifying the presentation of the overview of the intervention, a review of terminology and language used across the board, and a redesign of the social network mapping exercise to avoid focus on lack of support. We also received feedback on the length of the manual and how it may impact engagement, the process of planning and whether it was too complex for people’s cognitive abilities or educational background, and the need for a supporter as a criterion for the ability to participate in the intervention. It was decided that further testing would be conducted and these features kept as is for the feasibility study.

In further response to this feedback, it was also emphasized to facilitators during training that 1) if the person did not wish to write in the manual, the intervention provider or their supporter could fill in details instead, 2) activities could be completed verbally, or even omitted if necessary (eg, the activity being perceived as having limited utility or relevance by the person), 3) that the main aim of the intervention was for people to enact the plans the dementia adviser had facilitated them to create between sessions.

To inform the development of the PRIDE fidelity checklists,^54^ the resulting PRIDE manual was coded for BCTs by one researcher (HW) using the Behavior Change Technique Taxonomy Version 1 (see Table 2).^53^ The resulting BCTs are reported in this paper to further specify intervention content.

## Discussion

The PRIDE intervention and manual were developed within the framework described in the MRC guidelines.^12^ The structure and processes within the intervention are underpinned by the SOC model,^32^ social learning theory,^35^ and social network theory.^33^ Consultations were conducted with project stakeholders to obtain feedback on the first draft of the intervention and materials. Amendments related to presentation, ordering, language, content, and format were implemented. However, some aspects of feedback related to people’s cognitive abilities (eg, length of manual affecting motivation to engage, degree of planning needed to engage) and whether those who did not have a supporter could engage with the manual warrant further investigation.

### The development of PRIDE within the context of current postdiagnostic support services

Whilst early diagnosis has been a focus for health and social care services, there is a paucity of specific guidance or recommendations on the format or content of nonpharmacological postdiagnostic support packages.^55^ Various postdiagnostic initiatives have been devised including peer support, information provision, and adviser services. However, the availability of these is patchy with little robust evidence available on the associated benefits. A pilot project delivering person-centered pos-diagnostic support to people with early-stage dementia had favorable results, indicating that individualized support including social opportunities and provision of appropriate and timely information has the potential to positively impact people with dementia and may address service gaps.^56^ Furthermore, research from self-management programs for people with dementia,^26^ although limited, suggests they may address the current “care gap” supporting people living with early-stage dementia.^57^ In aiming to promote independence, and encourage engagement in beneficial activities, the PRIDE intervention addresses the impoverished postdiagnostic experiences reported by some memory clinic attendees^58^ whilst improving the development, application, and evidence for social science theory.

### Strengths and limitations

Work was undertaken to establish a theoretical basis for PRIDE in accordance with MRC guidance,^12^ which emphasizes the importance of applying a theoretical perspective in order to understand factors that influence behavior and select interventions that have an evidence base (eg, indirect evidence including similar interventions, biological plausibility, etc.). This may increase the likelihood that the intervention is appropriate for the behavior it seeks to target, and therefore increases its chance of being effective. In a review of online behavior change interventions, extensive use of theory was associated with larger effect sizes.^59^ This suggests that drawing on several robust theoretical models to form the design and content of the PRIDE intervention may augment its potential to elicit benefits.

Taking a manualized approach offers structure and allows for standardization of delivery, which can support providers to deliver the intervention as planned^60^ and enhance the quality of the intervention received.^61^ However, the role of the intervention providers will be to balance the structure provided by the manual with flexibility by personalizing content and communicating information from the manual to the person in an accessible way through discussions.^62^ In being directly focused on the person with dementia, PRIDE differs from other information-giving services and interventions currently available which have been criticized for catering for family members rather than the person themselves, furthering feelings of powerlessness and helplessness.^19^

At this stage, stakeholders provided prospective feedback on the intervention in principle. Although this was informative in shaping the program in its first iteration, neither the materials or intervention process was tested in practice; thus this feedback was somewhat limited. An advantage of holding consultations at an early stage prior to feasibility testing is that we were able to quickly canvas people’s opinions on the work as it was developing, remaining open to changing or retaining aspects of the program until further data had been gathered from more formal testing.^63^ The next stage of the study will seek to gather data on the feasibility of the intervention in practice, including experiences of barriers and facilitators, possible outcomes, suitability of the manual and proposed activities, suitability of dementia advisers as facilitators of the program, and structure of the intervention. This step will help to identify and safeguard against any issues, which may undermine the implementation^64^ and evaluation of the intervention.^12^

Although key stakeholders of the project were involved in the consultations, they were not equally represented in the sample. This reflected the opportunistic nature of recruitment, and that dementia advisers participating in group consultations were based within their organizations, which facilitated recruitment of greater numbers of dementia advisers than carers or especially people with dementia. In the feasibility phase, over90 people with dementia and their supporters will be recruited to test and feed-back on the intervention, plus a sub-sample will be asked to participate in post-study interviews. Key stakeholders will thus be more fully represented in this phase.^11^ To ensure that the care that is provided is fit for purpose and effectively addresses the need, the involvement of people with lived experience of dementia is essential in the development and evaluation of interventions and services.^65^,^66^

## Conclusion

The PRIDE intervention is designed for, and developed with, those with mild dementia who are aware of their diagnosis and retain the ability to read, write, and converse. It allows tailoring according to individual needs and circumstances, linked to an outcome-related set of activities. It is currently being tested in a multisite feasibility study.

The PRIDE intervention was developed for people in the early stages of dementia following the MRC framework.^12^ Consultations with stakeholders have played a key role in shaping the intervention and accompanying manual. The intervention seeks to provide information and support to help people with dementia to remain independent and engaged in activities based on the implementation of practical strategies derived from models including SOC, social learning theory, and social network theory. It also addresses the difficulties of receiving a diagnosis, changing relationships, and how they relate to making decisions and maintaining independence. Although designed to be used with the support of an intervention provider, the manual allows for individuals to use it between sessions, and indeed they are encouraged to keep using it beyond the formal sessions themselves.

The next phase of development includes a feasibility test of the intervention and manual in preparation for evaluation in a randomized trial, as well as the development of a web-based version of the manual. This will involve recruiting up to a further 80 individuals to take part in the intervention, testing outcome measures and study procedures, as well as further qualitative work on the acceptability of the intervention and manual and fidelity testing. Finally, a randomized controlled trial will be conducted to compare the intervention with treatment as usual. In the future, if feasible and effective, the PRIDE intervention could be implemented within postdiagnostic services provided by dementia adviser organizations, voluntary organizations, or NHS mental health trusts.
